# Increased cancer mortality in diabetic people treated with insulin: a register-based follow-up study

**DOI:** 10.1186/1472-6963-13-267

**Published:** 2013-07-09

**Authors:** Erja Forssas, Reijo Sund, Kristiina Manderbacka, Martti Arffman, Pirjo Ilanne-Parikka, Ilmo Keskimäki

**Affiliations:** 1 National Institute for Health and Welfare, (THL), Service System Department, P.O. Box 30, FI-00271 Helsinki, Finland; 2 The Finnish Diabetes Association, Tampere, Finland

**Keywords:** Diabetes mellitus, Excess mortality, Monitoring, Register study

## Abstract

**Background:**

The national 10-year Development Programme for the Prevention and Care of Diabetes (DEHKO) was launched in Finland in 2000. The program focused on improving early diagnosis of type 2 diabetes and preventing diabetes-related complications. The FinDM database was established for epidemiological monitoring of diabetes and its complications. This study monitors mortality trends among people with diabetes during the DEHKO programme.

**Methods:**

A database obtained from a compilation of several administrative national health registers was used to study mortality in people with diabetes in 1998–2007. Relative excess mortality between people with and without diabetes was analyzed using Poisson regression models.

**Results:**

The number of diabetic people in Finland increased by 66% from 1997 reaching 284 832 in 2007. Like among non-diabetic people, all-cause mortality decreased in people with diabetes. Overall excess mortality remained high in people with diabetes; in 2003–2007 RRs in the non-insulin treated was 1.82 for men and 1.95 for women and in the insulin treated 3.45 and 4.29, and excess coronary heart disease mortality in the insulin treated: RR was 4.71 in men and 7.80 in women. A striking result was mortality from neoplasms; an increase in mortality emerged in almost every age group of insulin treated women.

**Conclusion:**

Compared to non-diabetic people our monitoring showed declining excess mortality in non-insulin treated diabetic people mainly due to a decrease in mortality from cardiovascular diseases. For insulin treated, relative overall excess mortality remained unchanged and mortality from neoplasms increased among women.

## Background

The contribution of diabetes to the overall burden of disease has been rapidly increasing in the last twenty years. The increase in the numbers of diabetic people concerns both insulin and non-insulin dependent diabetes. This trend has been shown in Finland [1,2], as in many other countries [3-6]. While the prevalence of diabetes has increased worldwide and estimated to increase from 2.8% in 2000 to 4.4% in 2030 [7], the DIAMOND study showed an average 3% yearly increase in the incidence of type 1 diabetes in the 1990s worldwide [8].

While people with diabetes have earlier been shown to follow the non-diabetic population in terms of declining all-cause mortality in Finland [9,10] as well as in Great Britain and Norway [11,12], previous studies in Finland have shown that mortality among people with diabetes remains substantially higher compared to mortality among people without diabetes. In 1991–1996 the age-standardised coronary heart disease (CHD) mortality risk among people with diabetes was sixfold for women and over threefold for men, compared to persons without diabetes [13]. Excess mortality due to CHD has also been detected in other Finnish studies [14,15]. In addition, persons with diabetes had higher than average mortality for most other causes of death [14,15]. Similar results have also been observed in other countries. Mortality of people with diabetes has been reported to be higher than that of people without diabetes in all-cause mortality, in CHD mortality and in deaths from cancer [16-20].

The national 10-year Development Programme for the Prevention and Care of Diabetes (DEHKO) was launched in Finland in 2000 [21]. The program focused on improving early diagnosis of type 2 diabetes and preventing diabetes-related complications. The FinDM II database based on a national individual level linkage scheme of health insurance and care registers was established for epidemiological monitoring of diabetes and its complications [22]. The present study monitors changes in mortality among people with diabetes of varying ages in 1998–2007 in Finland. It also examines the extent of and changes in excess mortality according to different causes of death among insulin-treated and non-insulin-treated people with diabetes compared to people without diabetes.

## Methods

### The study population

This study is based on individual-level nationwide register data on persons with or without diabetes between January 1, 1998 and December 31, 2007 in Finland. Data on persons diagnosed for diabetes were obtained from the FinDM II database. FinDM II data linkages received permission to use study data from different statistical authorities (National Institute for Health and Welfare, Statistics Finland, The Social Insurance Institution in Finland). The data linkages were done by appropriate statistical authorities as required by Finnish data protection legislation. The research group received anonymised data in which individuals were not identifiable. FinDM II data included individuals with diabetes identified in different Finnish health registers: *1*) the register of individuals eligible for elevated reimbursement of medication for chronic conditions including diabetes, *2*) the prescription register including all reimbursed medicines purchased, *3*) the national hospital discharge registers including *3a*) all inpatient care and *3b*) outpatient hospital visits, *4*) the Causes of Death register, and *5*) the medical birth register. The follow-up data were obtained from the same registers. The Finnish personal identity codes unique to each resident used in all registers allowed deterministic record linkage within and between registers.

Individuals were considered to have diabetes since the first registration of diabetes in any of the registers. Women with gestational diabetes only were excluded. The type of diabetes was determined on the basis of prescription data. Persons with continuous insulin usage coded according to the ATC system (A10A) and no purchases of medication intended to increase pancreatic insulin secretion (sulphonylureas (ATC-code A10BB), sitagliptin/vildagliptin (A10BH), repaglinide (A10BX02), nateglinide (A10BX03) or exenatide (A10BX04)) were considered as insulin-treated (ITDM) and the others as non-insulin-treated (NITDM). The prevalent ITDM population increased from 33 259 in 1997 (the proportion of women 44.1%) to 39 575 in 2007 (women 42.1%). The corresponding increase for the prevalent NITDM population was from 138 337 (women 55.9%) to 245 257 (women 50.1%).

Tabulated data on Finnish population were obtained from the national population and causes of death statistics (yearly mean population and the number of deaths in each specific (three-digit ICD-10) cause of death stratified by sex and five-year age groups). The number of people without diabetes was 4 975 753 in 1997 and 5 015 652 in 2007. Corresponding tabulated data were derived from the individual level diabetes data stratified additionally by diabetes type. Numbers for the non-diabetes group were obtained by subtracting the diabetes group numbers from the total population numbers.

All analyses were performed using the registered main cause of death. Three-digit causes of death codes were classified into ten main categories according to the 10th revision of the International Statistical Classification of Diseases and Related Health Problems. All main categories containing enough cases were included in the analyses. Also more specific causes of death groups were analyzed, if there were enough cases and if changes between follow-up periods were observed.

### Statistical analyses

The study data consisted of tabulation of numbers of deaths and corresponding follow-up time by calendar time (1-year intervals), age (5-year intervals) at follow-up, diabetes status (ITDM, NITDM, no diabetes) and sex for each specific cause of death.

Mortality rates were modelled using Poisson regression model in which age and period midpoints were used as continuous independent variables, number of deaths as dependent variable, and logarithmic follow-up time in person years as offset. The functions of age and period were described using natural (cubic) splines [23]. Age-specific mortalities were chosen to represent situation in 2002–2003 and trends in mortality were examined using rate ratios relative to the year 1998.

To simplify the presentation, mortality was also studied in 15-year age groups per 1000 person years. Trends in mortality were scrutinised in two periods (1998–2002 and 2003–2007). Excess mortality was calculated as the difference between the mortality among people with diabetes aged 1–79 and the mortality among people without diabetes of the same age. The relative risks of excess mortality in two periods and the statistical significance of the interaction terms were obtained from Poisson regression models. The analyses were performed with the SAS (version 9.2; http://sas.com) and R (version 3.0.0; http://r-project.org) software packages.

## Results

**Figure 1:**
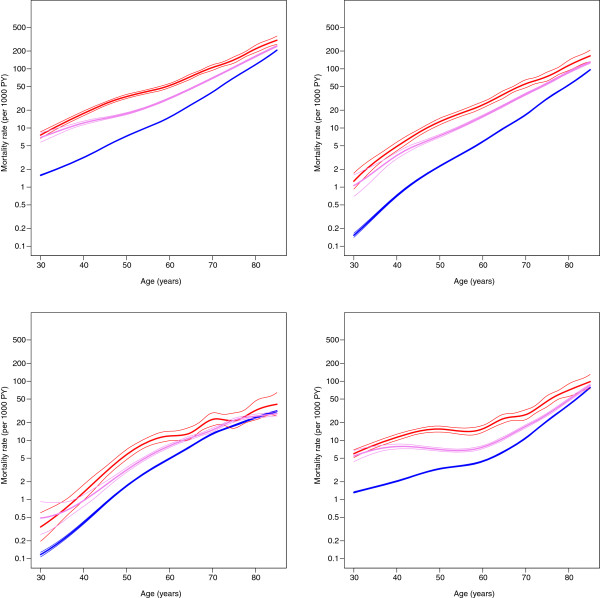
**Age**-**specific mortality (all-cause, circulatory, neoplasms and other diseases) among men in the reference years 2002– 2003.** Red is insulin-treated, violet non-insulin-treated and blue non-diabetic.

**Figure 2:**
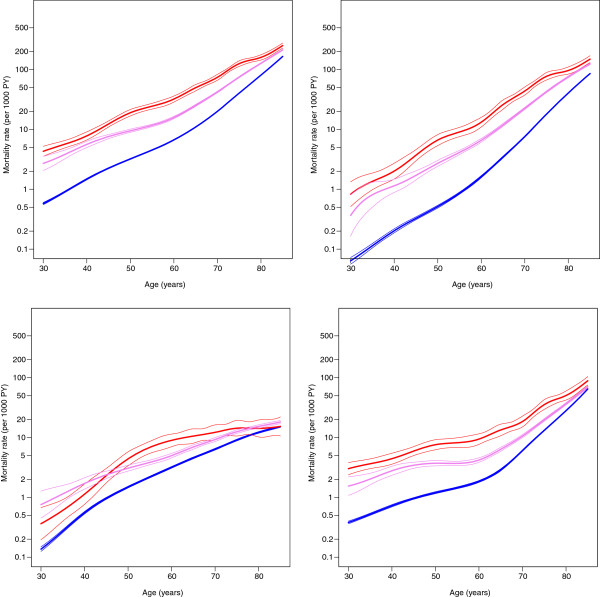
**Age**-**specific mortality (all**-**cause, circulatory, neoplasms and other diseases) among women in the reference years 2002– 2003.** Red is insulin-treated, violet non-insulin-treated and blue non-diabetic.

**Figure 3:**
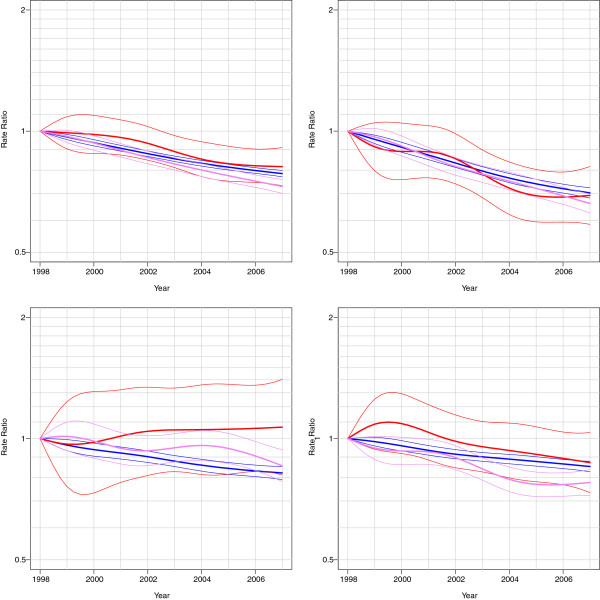
**Mortality trends among men (all**-**cause, circulatory, neoplasms and other diseases), relative to the reference year 1998.** Red is insulin-treated, violet non-insulin-treated and blue non-diabetic.

**Figure 4:**
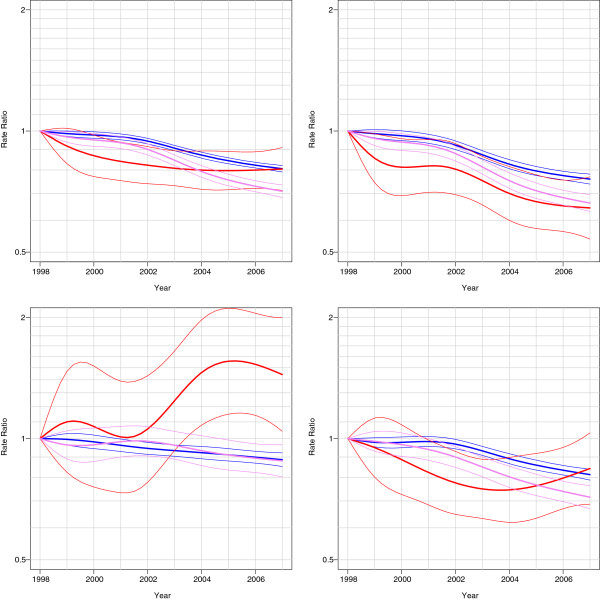
**Mortality trends among women (all**-**cause, circulatory, neoplasms and other diseases), relative to the reference year 1998.** Red is insulin-treated, violet non-insulin-treated and blue non-diabetic.

Age-specific mortality figures in 2002–2003 show that in all ages diabetic people have increased mortality rates compared to non-diabetic people (Figures 1 and 2). Decreasing mortality trends were found in people with and without diabetes in 1998–2007 except in mortality from neoplasms among insulin-treated persons (Figure 3 and 4). An increase in mortality from neoplasms emerged especially among women (Figure 4). From 1998–2002 to 2003–2007, the all-cause mortality among people with diabetes decreased in almost all age groups (Additional file 1: Table S1). In the same time the mortality from neoplasms increased almost in all age groups between the study periods. The increase was statistically significant among women aged 60–74. At the same time, statistically significant decrease was found in the mortality from neoplasms among people without diabetes. A decline in mortality rates from neoplasms and cardiovascular diseases (CVDs) was also found during the study periods among non-insulin-treated persons with diabetes. The decrease in CVD mortality was statistically significant in all age groups among non-insulin-dependent men with diabetes.

For people with insulin-treated diabetes, the excess overall mortality in 2003–2007 was more than threefold among men and fourfold among women compared to the people without diabetes (Additional file 2: Table S2). Excess mortality associated with CHD was almost eightfold among insulin-treated women and almost fivefold among men. No decrease in overall excess mortality from 1998–2002 to 2003–2007 was seen. A statistically significant increase between these two periods was observed in excess mortality from neoplasms both among insulin-treated men and women. The increase was more obvious among women, among whom neoplasm mortality increased especially with regard to malignancies of digestive organs, breast and urinary tract, or blood and lymphatic tissue.

For people with non-insulin-treated diabetes, the excess overall mortality in 2003–2007 was nearly twofold both among men and women compared to the people without diabetes (Additional file 3: Table S3). Excess mortality associated with CHD was over threefold among non-insulin-treated women and over twofold among men. A significant decrease in overall excess mortality from 1998–2002 to 2003–2007 was seen both among men and women. A statistically significant decrease between these two periods was mainly observed in excess mortality from CVD but among men also from respiratory diseases and diseases of the digestive system. A slightly, but statistically significant increase in excess mortality from neoplasm was observed among men.

## Discussion

We found a decline in the excess overall mortality rate for non-insulin-treated people with diabetes, especially with regard to CVD, compared to the people without diabetes between the periods from 1998–2002 to 2003–2007. However, the excess overall mortality rate showed no change for those with insulin-treated diabetes. Furthermore, a statistically significant increase in excess mortality from cancer was observed.

According to this study the all-cause mortality among people with diabetes decreased in almost all age groups from 1998 to 2007 as it has decreased among the general Finnish population [10]. Similar development has also been reported in other countries [11,12]. One explanation might be that the population with diabetes increases more rapidly due to more effective screening of type 2 diabetes, which was emphasized in the national diabetes prevention program in 2000–2010 in Finland. That might have brought people earlier to diagnosis and to better care. Although mortality among people with diabetes decreased, a significant excess mortality was still found. Some previous studies have also showed that the rates in all-cause mortality, in CHD mortality and in deaths from cancer are higher among people with diabetes than among people without diabetes [14-20].

To our knowledge, the present study is the first to examine the changes and associations in excess mortality from specific causes using the whole population together with all insulin- or non-insulin-treated individuals with diabetes. Tierney et al. [24] assessed changes in the excess mortality rate over time in the US for those with diabetes compared to those without diabetes using specific causes of deaths. They found that the excess mortality among men and women with diabetes decreased from 1992–1998 to 1999–2003 especially with regard to CVDs [24]. In spite of different study periods, these findings were consistent with our results concerning persons with non-insulin-treated diabetes who normally form the main part of the population with diabetes.

### Insulin-treated people with diabetes

The excess mortality rate for those with insulin-treated diabetes compared with those without diabetes remained stable except for increasing excess mortality from neoplasms.

Contrary to non-insulin-treated people with diabetes, the excess mortality associated with CVD did not decrease among insulin-treated people. In 2003–2007, for example the excess mortality from CHD was almost eightfold for women and almost fivefold for men with insulin-treated diabetes compared to people without diabetes. Our study is in line with earlier results describing the major impact of diabetes in increasing the risk of CHD mortality [13-16,18]. We found that excess CHD mortality was particularly high among insulin-treated people with diabetes. One reason for higher excess mortality among insulin-treated compared to non-insulin-treated individuals might be the longer duration of the disease. A previous study concerning insulin-dependent people with diabetes in Finland reported that the relative mortality, compared with the general population, was more strongly influenced by duration of diabetes than by age [25]. Also smoking prevalence might be one reason for higher CHD mortality among people with diabetes. A Finnish study conducted in the early 1990’s, discovered that young adults with diabetes smoked more than non-diabetic individuals [26].

Among insulin-treated people with diabetes, mortality from neoplasms increased almost in all age groups between the study periods. At the same time, mortality from neoplasms decreased among people without diabetes and showed also a decreasing trend among people with non-insulin treated diabetes. The increase in deaths from cancer was more obvious among insulin-treated women, among whom mortality increased especially with regard to cancer of digestive organs, breast and urinary tract, and lymphoid tissue and blood. Many studies have indicated that diabetes is associated with an increased risk of death from malignancies [19,20,27,28]. According to Barone and colleagues [17] there are several potential explanations for this. First higher insulin levels may contribute to increased tumor growth [29,30]. Another reason can be that cancer patients with diabetes might be treated less aggressively than those without diabetes [31]. Third, patients with diabetes may have poorer response to cancer treatment, including increased infection risk and intraoperative mortality [32]. However, it is difficult to explain the most obvious increase in cancer mortality among women with insulin-treated diabetes found in this study. Changes in treatment practices might have influenced in these worse outcomes. For example, in Sweden during 2006 and 2007, women using insulin glargine alone had an increased incidence risk of breast cancer as compared with women using types of insulin other than insulin glargine [33]. The same finding was also made in a Scottish study [34]. Further investigation is needed concerning the increased cancer mortality among insulin-treated diabetic individuals.

#### Non-insulin-treated people with diabetes

The excess mortality rate for persons with non-insulin-treated diabetes compared to those without diabetes appears to be clearly diminishing. The most obvious decrease found in excess mortality was that of CVD mortality. This may mainly have been due to improved preventive and acute treatment practices reported in an earlier study by the study group [35]. The proportion of people with diabetes using cholesterol lowering and antihypertensive drugs increased between 1997 and 2007 in Finland [35]. Another study compared the use of secondary preventive medication among newly diagnosed CHD patients with and without diabetes between 1997 and 2002 [36]. It was found that the use of a new class of antihypertensive drugs, angiotensin II receptor antagonists and ACE inhibitors was more common among people with diabetes compared to people without diabetes. The use of lipid lowering medication and β-blockers was almost similar in both groups. Also there has been found improvements in the proportion of people with type 2 diabetes achieving glycemic target levels in Finland [37]. Similar development has also been reported in a US study concerning the proportion of people with diagnosed diabetes achieving glycemic and LDL targets [38].

Our data were based on large national administrative datasets, namely hospital discharge and cause of death registers, and reimbursement for medication and prescription data derived from the Finnish Social Insurance Institution registries. The reliability of Finnish health registers has been evaluated good [39]. The reliability of the Finnish Cause of death registers is also considered good, due to fairly high overall autopsy rate [40]. Between 1996 and 2007 a medical or medico-legal autopsy was performed in about 30% of deaths [10].

A major strength of our study was the use of comprehensive national registers to follow the mortality for all treated individuals with diabetes in Finland over two five-year time period. In addition, we were able to compare different treatment groups of people with diabetes. Some methodological limitations still exist. Individuals treated with diet only were classified as non-diabetic individuals, if diabetes was not reported in any of the registers used. However, those treated with diet only are mainly among people aged over 65 [41]. The different treatment groups were not identified from the medical records but from medication and hospital discharge registers. However, those recorded insulin-treated have for the most type 1 diabetes and those with non-insulin treated type 2 diabetes. Despite these limitations this material is unique. Our data encompass the whole population of a country; all diabetic individuals receiving medication have been identified, and all cause of deaths in 1998–2007 have been registered and analyzed according to sex and diabetes treatment groups.

## Conclusions

The present study indicates that excess mortality has remained high among individuals with diabetes. Mortality among people with non-insulin-treated diabetes compared to the non-diabetic population has decreased especially with regard to CVDs, whereas no decrease among insulin-treated were seen. Among people with insulin-treated diabetes, significant increase was observed in excess mortality from cancer among women. Further investigation is needed concerning the increased cancer mortality among insulin-treated diabetic individuals.

## Competing interests

The authors declare that they have no competing interests.

## Authors’ contributions

EF designed and wrote the manuscript and performed the statistical analysis, RS participated in designing the study and wrote and reviewed the manuscript, KM wrote and reviewed the manuscript, MA helped analyzing the data and wrote and reviewed the manuscript, PIP reviewed the manuscript and contributed to the introduction and discussion, IK reviewed the manuscript and contributed to the discussion. All authors read and approved the final manuscript.

## Pre-publication history

The pre-publication history for this paper can be accessed here:

http://www.biomedcentral.com/1472-6963/13/267/prepub

## Supplementary Material

Additional file 1: Table S1 Mortality among people with and without diabetes in Finland in 1998–2002 and 2003–2007 (per 1000 person years). Click here for file

Additional file 2: Table S2 Age standardized mortality (95% confidence interval) of insulin-treated people with diabetes aged 1–79 compared to people without diabetes (=1.00) by cause of death in 1998–2002 and 2003-2007. ^a^Significance of the difference between age adjusted excess mortality in 1998–2002 and in 2003–2007 [interaction between diabetes status (insulin treated diabetic people vs non-diabetic people) and two time periods], * = p < 0.05, ** = p < 0.01, *** = p < 0.001; otherwise non-significant. Click here for file

Additional file 3: Table S3 Age standardized mortality (95% confidence interval) of non-insulin-treated people with diabetes aged 1–79 compared to people without diabetes (=1.00) by cause of death in 1998–2002 and 2003-2007. ^a^Significance of the difference between age adjusted excess mortality in 1998–2002 and in 2003–2007 [interaction between diabetes status (non-insulin treated diabetic people vs non-diabetic people) and two time periods], * = p < 0.05, *** = p < 0.001; otherwise non-significant. Click here for file
